# A System Review and Meta-Analysis of Canaloplasty Outcomes in Glaucoma Treatment in Comparison with Trabeculectomy

**DOI:** 10.1155/2017/2723761

**Published:** 2017-04-30

**Authors:** Bing Zhang, Jie Kang, Xiaoming Chen

**Affiliations:** Department of Ophthalmology, West China Hospital, Sichuan University, 37 Guo Xue Xiang, Chengdu 610041, China

## Abstract

*Purpose.* This system review studied the efficiency and safety of canaloplasty (CP) and compared the outcomes between CP and trabeculectomy (TE). *Methods*. Literatures were searched in PubMed and EMBASE. The meta-analysis was conducted on the postoperative outcomes in CP and then on the differences of outcomes between CP and TE. *Results*. In the meta-analysis, IOP decreased by 9.94 (95% CI 8.42 to 11.45) mmHg with an average AGM reduction of 2.11 (95% CI 1.80 to 2.42) one year after CP. The IOP reduction was significantly higher after TE than after CP, with an average difference of 3.61 (95% CI 1.69 to 5.53) mmHg at 12 months postoperationally. For complications, the incidence of hyphema was significantly higher in CP and the Descemet membrane detachment was just reported in CP, with an incidence of 3%. However, the incidence was significantly lower in CP of hypotony and of choroidal effusion/detachment. Meanwhile, suprachoroidal hemorrhage and bleb needling were only reported in TE. *Conclusions*. CP was less effective in IOP reduction than TE, but CP was able to achieve similar postoperative success rates and reduce the number of AGMs likewise. CP was also associated with lower incidence of complications. More high-quality researches are needed in the future to verify our findings in this system review.

## 1. Introduction

Intraocular pressure (IOP) is by now the most proven treatable factor in glaucoma, and lowering IOP has long been associated with slowing the damages by glaucoma [1, 2]. Trabeculectomy (TE), since firstly being introduced in the 1960s, has remained the standard surgery of IOP control in glaucoma [3]. However, the relatively high incidence of complications of TE [3, 4] has encouraged the development of new surgery methods.

Canaloplasty (CP) was a nonpenetrating surgery (NPS) performed with a microcatheter (iTrack; iScience Surgical Corp.). During CP, the Schlemm canal is expanded circumferentially injecting a small amount of high-weight molecular viscoelastic agent with iTrack and then a suture loop is placed to apply tension to the trabecular meshwork when iTrack is retracted [5]. CP has been performed as one major NPS in open-angle glaucoma treatment for years. However, no system review of CP to evaluate its efficiency and complications in the treatment of glaucoma has been published by now as far as we know. This study firstly made a system review of CP and then compared the efficiency and complications between CP and TE.

## 2. Methods

This research was accorded to a predetermined protocol based on the Cochrane Handbook for Systematic Reviews of Interventions [6]. Review board approval was not required as no patients were enrolled.

### 2.1. Eligibility Criteria

Studies which met the following criteria were considered eligible: (1) participants diagnosed with glaucoma regardless of age, sex, or race, studies limited in patients with another failed antiglaucoma surgery were excluded; (2) interventions, included but not limited to CP, with or without phacoemulsification; (3) research types, both prospective and retrospective studies, excluding case reports and reviews; (4) outcomes: included but not limited to IOP, the follow-up was at least 6 months; (5) for literatures with overlapping data, only the one with the largest sample and then the longest follow-up was included.

### 2.2. Search Strategies

PubMed and EMBASE were searched for literatures published up to April 1, 2016, in any language with the following strategies: ((circumferential OR 360) AND (viscocanalostomy OR viscodilat^∗^)) OR canaloplasty. No unpublished studies were searched. After removing duplicate records, two reviewers (Bing and Jie) independently decided whether a study met the inclusion criteria; exclusion reasons were given to every literature not included.

### 2.3. Outcome Measures

The primary outcomes were the changes in IOP and the number of antiglaucoma medications (AGMs). The secondary outcomes were the complete and qualified successful rates and the incidence of adverse events. A complete success is defined as that a confirmed IOP is less than a given level without any AGMs; a qualified success is defined as that a confirmed IOP is less than a given level with or without AGMs [7]. The outcomes and research information were extracted by two researchers (Bing and Jie) independently.

### 2.4. Statistical Analysis

The meta-analysis was conducted with the software Review Manager V5.2 (Cochrane Collaboration). The changes in IOP and AGMs after CP were meta-analyzed in three subgroups, CP standalone, CP with phacoemulsification (PCP), and CP mixed (the former two subgroups mixed in the original papers). The mean between-group difference (MeD) of reductions in IOP and AGMs and the odds ratios (ORs) of the success rates and the incidence of complications were analyzed between CP and TE in two subgroups, standalone CP versus standalone TE and PCP versus phacotrabeculectomy (PTE). The random effects model was applied in most cases as the heterogeneity was considered present in the enrolled studies for clinical and study differences.

### 2.5. Sensitivity Analysis, Publication Bias Analysis, and Quality Assessment

Sensitivity analysis was performed for IOP and AGM reductions in CP by deleting the following subgroups: (a) all retrospective studies and (b) studies with small weight (less than the average weight). The publication bias was analyzed with the asymmetry of funnel plot [8]. Neither sensitivity analysis nor funnel plot analysis was conducted in the meta-analysis outcomes between CP and TE, with only 6 literatures enrolled. The quality assessment was performed in the meta-analysis between CP and TE; 1 randomized controlled trial (RCT) was analyzed with the risk of bias table according to the Cochrane Handbook for Systematic Reviews of Interventions [6] and the other 5 non-RCTs were analyzed with the Newcastle-Ottawa Quality Assessment Scale [9]. All analyses in this part were performed by two researchers (Bing and Jie) independently.

## 3. Results

### 3.1. Literatures Selection and Characteristics of the Enrolled Studies

**Figure 1:**
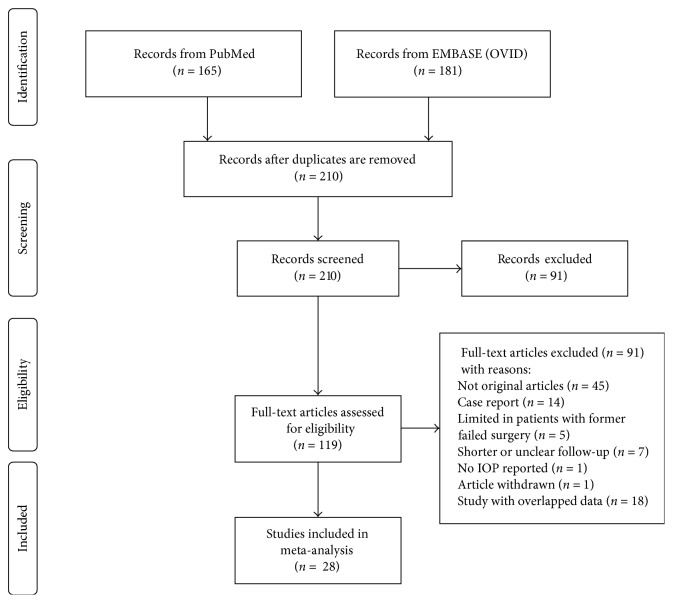
Flow diagram for study selection.

210 records returned from the initial literature search after deduplicating 136 records. 28 records were included in the quantitative analysis, and the other 182 did not meet the eligibility criteria as the process shown in Figure 1. An update of the literature search was made in February 3, 2017, and no new study was enrolled to the quantitative analysis.  Table 1 shows the descriptions of the enrolled 28 literatures. 1498 eyes were enrolled at baseline totally, 78% of which were diagnosed with primary open-angle glaucoma (POAG). The average age of patients at baseline was 62.7 ± 15.4 years in the independent CP subgroup and 71.2 ± 9.6 years in the PCP subgroups. The average baseline IOP was 25.1 ± 8.5 mmHg with 3.04 ± 1.18 AGMs in CP standalone subgroup, and the mean baseline IOP was 20.7 ± 6.4 mm Hg with 2.23 ± 1.14 AGMs in the PCP subgroup.

### 3.2. The Efficiency of CP

The reduction of IOP in all subgroups at 6 months was 10.69 (95% CI 8.96 to12.43) mmHg with 2.03 (95% CI 1.69 to 2.37) less AGM use and at 12 months was 9.94 (95% CI 8.42 to 11.45) mmHg with 2.11 (95% CI 1.80 to 2.42) less AGM use.  Table 2 shows more information of the meta-analysis results (detailed information and the forest plots in Online Resource Figures 1–4 in Supplementary Material available online at https://doi.org/10.1155/2017/2723761).

### 3.3. The Complications of CP

Table 3 summarizes the incidence of complications of CP (included PCP). The most common one was hyphema; hyphema (blood layer > 1 mm) could be observed in about every four enrolled eyes (24.9%). The incidence of the other complications in CP is shown in Table 3.

### 3.4. Comparison of CP and TE

#### 3.4.1. Reductions in IOP and AGMs

**Figure 2:**
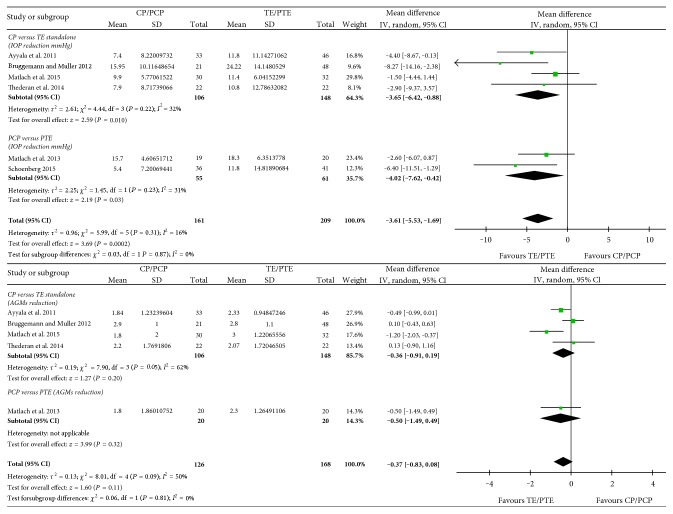
Comparison of the reductions in IOP and antiglaucoma medications (AGMs) between canaloplasty (CP) and trabeculectomy (TE) (PCP = phacocanaloplasty, PTE = phacotrabeculectomy).

The meta-analysis results of the MeDs between TE and CP of the reductions in IOP and AGMs at 12 months after surgery are shown in Figure 2() (data at 6 months used in Bruggemann and Muller [13]). TE was more efficient in IOP control than CP, with a MeD of 3.61 (95% CI, 1.69~5.53) mmHg. However, no significant difference was found in the reduction of AGMs between CP and TE, with a MeD of −0.37 (95% CI −0.83~0.08).

#### 3.4.2. The Success Rates

**Figure 3:**
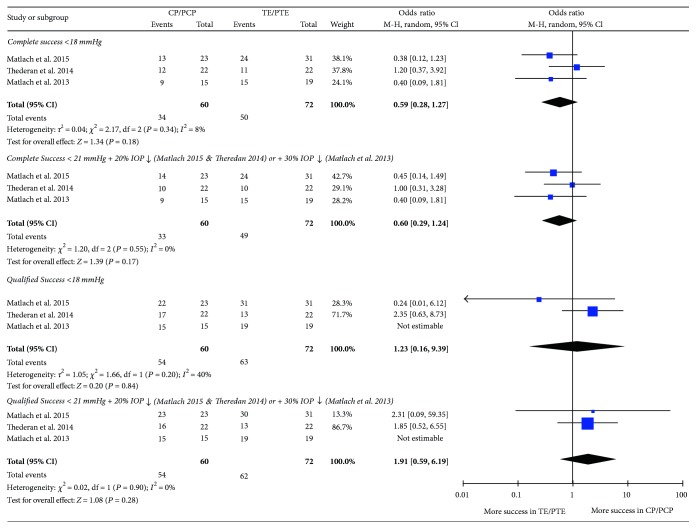
Comparison of success rates between canaloplasty (CP) and trabeculectomy (TE) (independent CP versus TE in Matlach et al. [22] and Thederan et al. [26]; PCP versus PTE in Matlach [31]; PCP = phacocanaloplasty, PTE = phacotrabeculectomy).

The complete and qualified success rates were compared between CP and TE at 12 months in three studies [22, 26, 31] as shown in Figure 3. No significant difference in the success rates was found between CP and TE in all comparisons shown in Figure 3.

#### 3.4.3. The Complications

**Figure 4:**
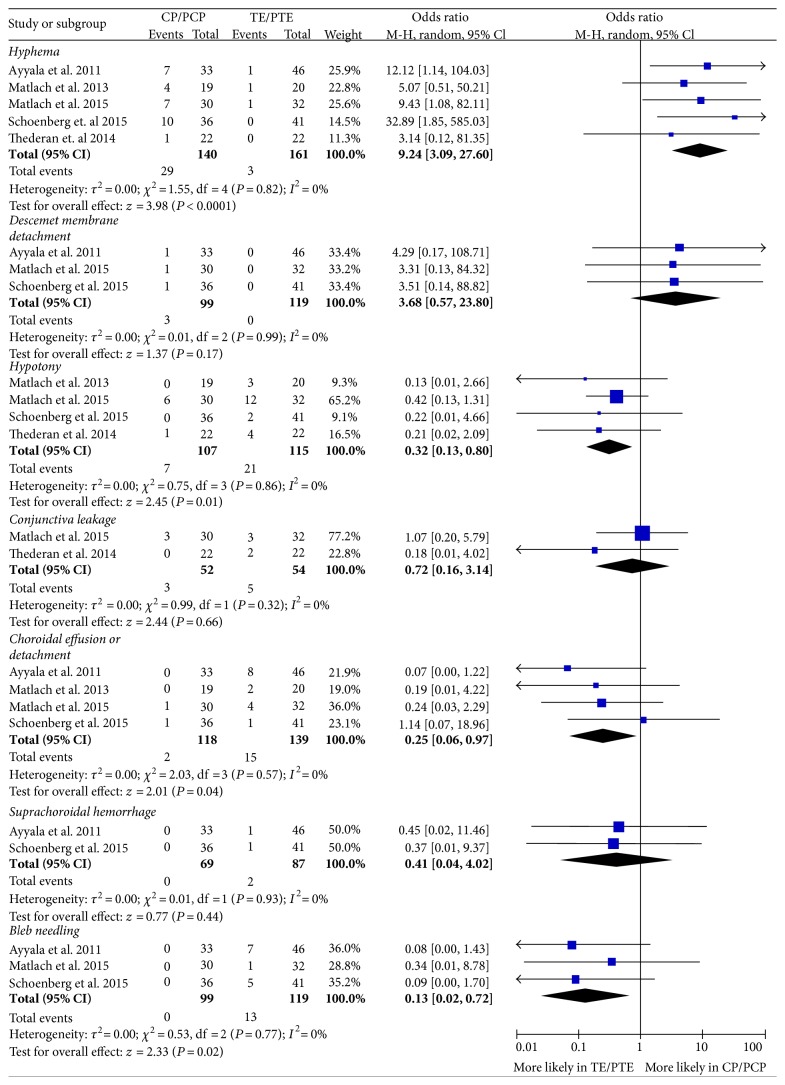
Comparison of complications between canaloplasty (CP) and trabeculectomy (TE).

The complications were compared between CP and TE in five researches [10, 22, 26, 31, 33] with ORs as shown in Figure 4. The hyphema was more prevalent in CP with an OR of 9.24 (95% CI, 3.09 to 27.60). The Descemet membrane detachment was only observed in CP with a reported incidence of 3%. The suprachoroidal hemorrhage and bleb needling were only reported in TE with incidences of 2.3% and 10.9%, respectively. TE was with significantly higher incidences in hypotony and the choroidal effusion/detachment. No significant difference was found in the incidence of conjunctiva leakage (OR 0.72, 95% CI 0.16 to 3.14).

#### 3.4.4. Sensitivity Analysis

In sensitivity analysis, the difference between before and after removing all retrospective studies in the IOP reductions was 14% at 6 months and 17% at 12 months; the corresponding difference in the AGM reductions was 5% at 6 months and 5% at 12 months. The difference between before and after removing the small weight studies in the IOP reduction was −3% at 6 months and −9% at 12 months; the corresponding change in the AGM reduction was 4% at 6 months and 0% at 12 months (detailed data in the Online Resource Table 1). The publication bias analysis and the quality assessment were given in the discussion part.

## 4. Discussion

As far as we know, this was the first system review of CP in glaucoma control. At one year after CP, IOP decreased by 9.94 (95% CI 8.42 to 11.45) mmHg with 2.11 (95% CI 1.80 to 2.42) less AGM use. TE was shown to be more efficient in IOP control than in CP, with 3.61 (95% CI, 1.69 to 5.53) mmHg more IOP reduction at 12 months postoperationally. Our finding confirmed the conclusion of Rulli's meta-analysis that NPS was less effective than TE in decreasing IOP and also supported the opinion that canal surgery was less effective than TE in IOP control [3]. However, no significant difference between CP and TE was found in the AGM reduction and in the complete or qualified success rates at 1 year after surgery. CP was able to achieve similar postoperative success rates and reduce the number of the AGMs likewise.

In CP, hyphema was the most prevalent complication and nearly one in every four eyes would experience ≥1 mm hyphema. However, studies showed postoperative hyphema might indicate a better IOP control after CP, as it might be associated with restored aqueous outflow system [17, 37]. Descemet membrane detachment (DMD) was not a common complication after CP with an incidence of 3% in this review, and most DMD could be resolved without long-lasting sequelae and its risk might be decreased by avoiding excessive injection into the Schlemm canal during viscodilation [38]. However, one 86-year-old man was reported to develop keratoplasty-needing corneal decompensation from DMD after CP [38].

Comparing complications between CP and TE, hyphema was more common in CP, but might be a good indicator for CP as mentioned before. DMD was only reported in CP and could be related to the Schlemm canal injection [38]. Hypotony and choroidal effusion/detachment were more common in TE than in CP, which was in agreement with former research [3]. In TE, hypotony might be the result of high filtration, while choroidal effusion often occurred with hypotony. Suprachoroidal hemorrhage, a severe complication, was only reported in TE with an incidence of 2.3% in this review [10, 33]. Bleb needling was an intervention reported in 10.9% of eyes after TE and in no eyes after CP as an NPS. To sum up, TE was associated with more complications than CP.

CP was reported with higher patient satisfaction than TE in a multiquestionnaire study conducted two years after surgery [39]. Another cost-effectiveness study indicated that although the surgery fee was higher in CP, the longer hospitalization, higher readmission rates, and more frequent postoperative interventions of TE displayed opportunity costs [13], which should be taken into consideration.

In the sensitivity analysis, the outcomes of the meta-analysis of the reductions in IOP and AGMs after CP were robust, especially after removing small weight studies. The publication bias might exist with missing studies on the left hand side of the funnel plots of IOP reduction at 6 months (Online Resource Figure 5) and 12 months (Online Resource Figure 6) after surgery [8]. In the quality assessment of the enrolled 6 studies comparing CP with TE, the RCT [22] showed higher risk of performance, detection, and attrition bias (Online Resource Figure 7). For the other 5 retrospective studies, the main problem existed in the selection of the controls (Online Resource Table 2), as clinical heterogeneity might exist between the CP cases and the TE controls.

One main limitation of this system review was the quality of the enrolled studies. Without limitation of research types as few RCTs were available, 18 of the 28 eligible literatures were retrospective studies, a study type with relatively low evidence level. Moreover, not many studies about the comparison between CP and TE were available, and only 1 was an RCT and the other 5 were retrospective. Another limitation was no unpublished data were enrolled in this review while the funnel plot indicated publication bias might exist.

## 5. Conclusion

CP was less effective in IOP reduction than TE, but CP was able to achieve similar postoperative success rates and reduce the number of the AGMs likewise. CP was also associated with lower incidence of complications and was reported with higher patient satisfaction. More high-quality studies, especially properly designed RCTs, are needed to verify our findings in this system review.

## Supplementary Material

A systemic review and meta-analysis of canaloplasty outcomes in glaucoma treatment, in comparison with trabeculectomy.

## Figures and Tables

**Table 1:** Characteristics and baseline information of included literatures.

	Study type	Age, mean (SD)	Male%	Eyes at baseline	POAG%	Baseline IOP, mean (SD)	Baseline AGMs, mean (SD)
*Canaloplasty standalone*							
Ayyala et al. [10]	Retrospective	68.3 (10.0)	52	33	NA	21.2 (6.6)	2.5 (0.8)
Barnebey [11]	Retrospective	68.2 (13.1)	55	20	100	23.4 (4.3)	2.2 (1.2)
Brandao et al. [12]	Prospective	71.3 (7.3)	41	19	74	24.6 (5.3)	2.5 (0.8)
Bruggemann and Muller [13]	Retrospective	62.7 (NA)	52	21	100	28.8 (9.6)	2.9 (1.0)
Brusini [14]	Prospective	63.5 (14.0)	NA	214	74	29.4 (7.9)	3.3 (0.9)
Gandolfi et al. [15]	Retrospective	NA	67	24	67	26.0 (4.0)	2.7 (3.3)
Grieshaber et al. [16]	Prospective	49.8 (15.7)	43	60	100	45.0 (12.1)	NA
Grieshaber et al. [17]	Prospective	70.8 (8.4)	49	47	100	26.8 (5.2)	2.8 (0.5)
Grieshaber et al. [18]	Prospective	71.8 (8.8)	41	22	100	27.1 (5.3)	2.9 (0.6)
Kalin-Hajdu et al. [19]	Retrospective	40.0 (19.2)	NA	19	0	30.4 (8.4)	3.7 (0.8)
Lewis et al. [20] (CP)	Prospective	67.6 (11.6)	47	103	89	23.5 (4.5)	1.9 (0.8)
Lommatzsch et al. [7]	Retrospective	40.7 (21.8)	25	14	0	27.1 (12.3)	2.7 (1.1)
Lopes-Cardoso et al. [21] (CP)	Prospective	73.4 (6.0)	66	11	37	24.5 (5.1)	3.4 (0.5)
Matlach et al. [22]	RCT	66.5 (11.3)	60	30	43	23.7 (5.1)	2.6 (1.6)
Matthaei et al. [23] (CP)	Retrospective	65.2 (12.4)	46	33	87	18.5 (6.3)	2.3 (1.2)
Moelle et al. [24]	Retrospective	62 (9)	42	26	54	21.1 (5.8)	2.5 (1.8)
Seuthe et al. [25]	Retrospective	66.7 (11.4)	NA	417	86	20.9 (3.5)	3.5 (0.9)
Thederan et al. [26]	Retrospective	72.9 (5.2)	64	22	68	23.7 (7.6)	3.1 (1.2)
Voykov et al. [27]	Retrospective	60 (11)	45	20	75	25.7 (6.6)	3.4 (0.5)
Wang et al. [28]	Retrospective	39.1 (13.8)	77	17	NA	24.7 (8.7)	2.4 (1.7)
Xin et al. [29]	Prospective	38 (12.8)	65	17	100	29.9 (8.2)	2.9 (0.9)
*Canaloplasty with phacoemulsification*							
Arthur et al. [30]	Retrospective	76.1 (8.3)	56	32	84	18.2 (5.1)	1.3 (0.7)
Lewis et al. [20] (PCP)	Prospective	67.6 (11.6)	47	30	89	23.5 (5.2)	1.5 (1.0)
Lopes-Cardoso et al. [21] (PCP)	Prospective	73.4 (6.0)	66	24	37	19.8 (6.8)	3.3 (0.5)
Matlach et al. [31]	Retrospective	72.9 (5.7)	47	19	47	28.3 (4.1)	2.8 (1.1)
Matthaei et al. [23] (PCP)	Retrospective	65.2 (12.4)	46	13	87	17.5 (4.2)	2.5 (1.3)
Rekas et al. [32]	RCT	74.6 (8.9)	59	29	83	19.0 (6.9)	2.6 (0.7)
Schoenberg et al. [33]	Retrospective	66.8 (8.5)	67	36	94	19.5 (5.7)	NA
*Canaloplasty with or without* *phacoemulsification, mixed results*							
Alobeidan and Almobarak [34]	Retrospective	53.4 (15.0)	60	105	67	19.0 (6.7)	2.6 (1.3)
Fujita et al. [35]	Retrospective	71.7 (8.5)	NA	11	100	23.4 (5.5)	2.8 (0.6)
Rekas et al. [36]	Prospective	69.3 (11.4)	40	10	90	19.1 (3.4)	2.2 (1.1)

AGM: antiglaucoma medication; CP: independent canaloplasty subgroup in corresponding literature; NA: data not available; PCP: phacocanaloplasty subgroup in corresponding literature; POAG: primary open-angle glaucoma; RCT: randomized controlled trials.

**Table 2:** Meta-analysis outcomes of reductions in IOP and antiglaucoma medications (AGMs) at 6 and 12 months postoperationally.

		IOP reduction (95% CI) mmHg	AGMs, reduction (95% CI)
Standalone canaloplasty	6 months	12.01 (9.77, 14.24)	2.01 (1.51, 2.50)
	12 months	11.38 (9.43, 13.34)	2.16 (1.79, 2.53)
Phacocanaloplasty	6 months	8.32 (5.36, 11.27)	2.03 (1.36, 2.70)
	12 months	8.14 (4.83, 11.46)	2.04 (1.15, 2.92)

**Table 3:** Incidence of complications of canaloplasty.

Complications	Incidence% (events/pooled eyes)
Hyphema (blood layer > 1 mm)	24.9 (304/1221)
Hypotony < 5 mmHg	8.6 (94/1091)
Descemet membrane detachment	3.1 (37/1185)
Detectable conjunctival bleb	1.9 (17/899)
