# Supplementary material for: A System Review and Meta-Analysis of Canaloplasty Outcomes in Glaucoma Treatment in Comparison with Trabeculectomy
Source: J Ophthalmol. 2017 Apr 30;2017:2723761. doi: 10.1155/2017/2723761 (PMC5429958; doi:10.1155/2017/2723761)
Supplement: Supplementary file 1 — A systemic review and meta-analysis of canaloplasty outcomes in glaucoma treatment, in comparison with trabeculectomy. [file 2723761.f1.pdf]

A systemic review and meta-analysis of canaloplasty outcomes in glaucoma treatment, in comparison with trabeculectomy---Supporting Material

	Baseline			6 month postoperation				Mean Difference
Study or Subgroup	Mean	SD	Total	Mean	SD	Total	Weight	IV, Random, 95% CI
**8.1.1 Canloplasty**								
Ayyala 2011	21.2	6.6	33	12.1	4	32	4.3%	9.10 [6.46, 11.74]
Barnebey 2013	23.4	4.3	20	0	0	0		Not estimable
Brandao 2015	24.6	5.29	19	13.2	3.4	19	4.3%	11.40 [8.57, 14.23]
Bruggemann 2012	28.75	9.57	21	12.8	3.28	21	3.7%	15.95 [11.62, 20.28]
Brusini 2014	29.4	7.9	214	17.3	4.8	194	4.7%	12.10 [10.84, 13.36]
Gandolfi 2016	26	4	24	0	0	0		Not estimable
Grieshaber 2010	45	12.1	60	15.4	5.4	57	4.1%	29.60 [26.23, 32.97]
Grieshaber 2013	26.8	5.2	47	13.55	2.23	47	4.6%	13.25 [11.63, 14.87]
Grieshaber 2016	27.1	5.3	22	13.6	1.6	22	4.4%	13.50 [11.19, 15.81]
Kalin-Hajdu 2014	30.4	8.4	19	15.2	7.3	19	3.4%	15.20 [10.20, 20.20]
Lewis 2011	23.5	4.5	103	16.1	3.4	86	4.7%	7.40 [6.27, 8.53]
Lommatzsch 2016	27.07	12.28	14	0	0	0		Not estimable
Lopes-Cardoso 2013	24.5	5.1	11	12.8	3.2	8	3.9%	11.70 [7.96, 15.44]
Matlach 2015	23.7	5.1	30	13.8	2.6	24	4.5%	9.90 [7.80, 12.00]
Matthaei 2011	18.5	6.3	33	12.1	3	23	4.4%	6.40 [3.93, 8.87]
Moelle 2014	21.1	5.8	26	14.25	4.3	25	4.3%	6.85 [4.06, 9.64]
Seuthe 2016	20.86	3.544	417	0	0	0		Not estimable
Thederan 2014	23.7	7.6	22	15.7	4.9	22	3.9%	8.00 [4.22, 11.78]
Voykov 2015	25.7	6.6	20	0	0	0		Not estimable
Wang 2014	24.7	8.7	17	14.9	2.5	17	3.7%	9.80 [5.50, 14.10]
Xin 2016	29.9	8.2	17	15.7	8.3	17	3.2%	14.20 [8.65, 19.75]
**Subtotal (95% CI)**			**1189**			**633**	**66.0%**	**12.01 [9.77, 14.24]**
Heterogeneity: Tau² = 18.16; Chi² = 207.50, df = 15 (P < 0.00001); I² = 93%								
Test for overall effect: Z = 10.52 (P < 0.00001)								
**8.1.2 Phacocanaloplasty**								
Arthur 2014	18.2	5.1	32	13.6	3	26	4.5%	4.60 [2.49, 6.71]
Lewis 2011	23.5	5.2	30	12.8	2.9	25	4.5%	10.70 [8.52, 12.88]
Lopes-Cardoso 2013	19.8	6.8	24	11.8	4.6	11	3.9%	8.00 [4.15, 11.85]
Matlach 2013	28.3	4.1	19	14.2	4.6	17	4.2%	14.10 [11.24, 16.96]
Matthaei 2011	17.5	4.2	13	10.7	2.8	9	4.2%	6.80 [3.87, 9.73]
Rekas 2015	19	6.9	29	13.2	3.4	29	4.3%	5.80 [3.00, 8.60]
Schoenberg 2015	19.5	5.7	36	0	0	0		Not estimable
**Subtotal (95% CI)**			**183**			**117**	**25.6%**	**8.32 [5.36, 11.27]**
Heterogeneity: Tau² = 11.58; Chi² = 36.28, df = 5 (P < 0.00001); I² = 86%								
Test for overall effect: Z = 5.52 (P < 0.00001)								
**8.1.3 Canloplasty+Phacocanaloplasty**								
Alobeidan 2015	19.04	6.757	105	12.73285714	3.19	105	4.6%	6.31 [4.88, 7.74]
Fujita 2011	23.4	5.5	11	14	4.4	11	3.8%	9.40 [5.24, 13.56]
Rekas 2016	19.1	3.4	10	0	0	0		Not estimable
**Subtotal (95% CI)**			**126**			**116**	**8.4%**	**7.21 [4.45, 9.97]**
Heterogeneity: Tau² = 2.26; Chi² = 1.90, df = 1 (P = 0.17); I² = 47%								
Test for overall effect: Z = 5.13 (P < 0.00001)								
**Total (95% CI)**			**1498**			**866**	**100.0%**	**10.69 [8.96, 12.43]**
Heterogeneity: Tau² = 16.36; Chi² = 288.33, df = 23 (P < 0.00001); I² = 92%								
Test for overall effect: Z = 12.07 (P < 0.00001)								
Test for subgroup differences: Chi² = 8.05, df = 2 (P = 0.02), I² = 75.2%								

Mean Difference
IV, Random, 95% CI

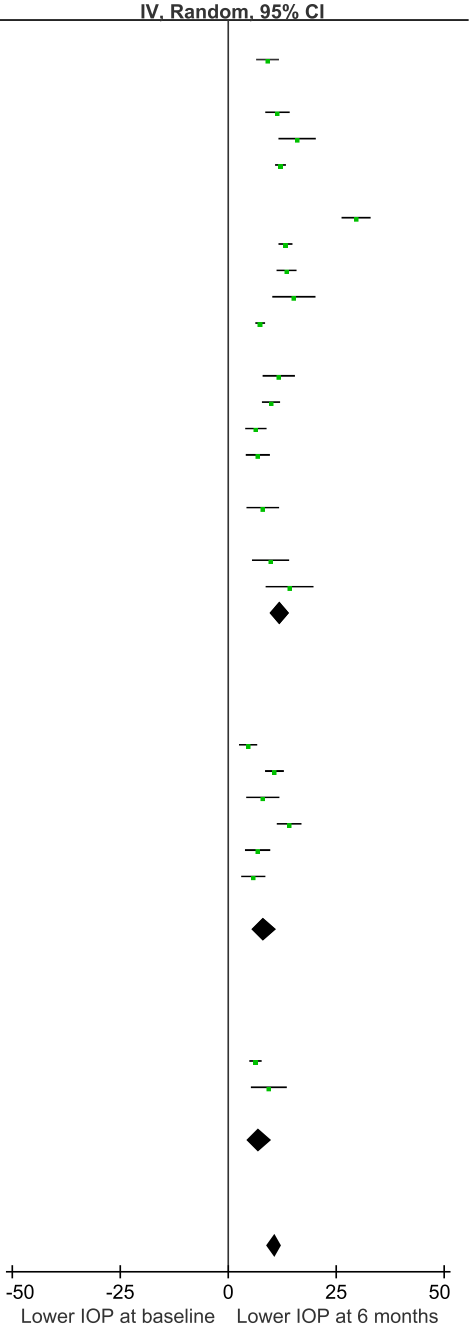

S Fig.1 IOP reduction in canaloplasty at 6 months after surgery

Study or Subgroup	Baseline Mean	Baseline SD	Baseline Total	12 month postoperation Mean	12 month postoperation SD	12 month postoperation Total	Weight	Mean Difference IV, Random, 95% CI
**8.3.1 Canloplasty**								
Ayyala 2011	21.2	6.6	33	13.8	4.9	33	3.7%	7.40 [4.60, 10.20]
Barnebey 2013	23.4	4.3	20	13.4	4.3	20	3.7%	10.00 [7.33, 12.67]
Brandao 2015	24.6	5.29	19	13.8	2.65	19	3.7%	10.80 [8.14, 13.46]
Bruggemann 2012	28.75	9.57	21	0	0	0		Not estimable
Brusini 2014	29.4	7.9	214	16.8	4.2	144	4.1%	12.60 [11.34, 13.86]
Gandolfi 2016	26	4	24	0	0	0		Not estimable
Grieshaber 2010	45	12.1	60	15.4	5.2	54	3.5%	29.60 [26.24, 32.96]
Grieshaber 2013	26.8	5.2	47	12.8	1.7	38	4.0%	14.00 [12.42, 15.58]
Grieshaber 2016	27.1	5.3	22	13.1	2.2	22	3.8%	14.00 [11.60, 16.40]
Kalin-Hajdu 2014	30.4	8.4	19	15.4	6.7	18	2.9%	15.00 [10.12, 19.88]
Lewis 2011	23.5	4.5	103	16.1	3.9	91	4.1%	7.40 [6.22, 8.58]
Lommatzsch 2016	27.07	12.28	14	16.35	6.33	14	2.1%	10.72 [3.48, 17.96]
Lopes-Cardoso 2013	24.5	5.1	11	13.5	1	4	3.6%	11.00 [7.83, 14.17]
Matlach 2015	23.7	5.1	30	13.8	2.7	23	3.9%	9.90 [7.77, 12.03]
Matthaei 2011	18.5	6.3	33	12.4	3.1	5	3.4%	6.10 [2.64, 9.56]
Moelle 2014	21.1	5.8	26	0	0	0		Not estimable
Seuthe 2016	20.86	3.544	417	13.519632	2.58	326	4.2%	7.34 [6.90, 7.78]
Thederan 2014	23.7	7.6	22	15.8	4.27	21	3.4%	7.90 [4.24, 11.56]
Voykov 2015	25.7	6.6	20	15.5	3.8	19	3.5%	10.20 [6.84, 13.56]
Wang 2014	24.7	8.7	17	14.7	2.1	17	3.2%	10.00 [5.75, 14.25]
Xin 2016	29.9	8.2	17	17.8	2.7	17	3.2%	12.10 [8.00, 16.20]
**Subtotal (95% CI)**			**1189**			**885**	**64.0%**	**11.38 [9.43, 13.34]**
Heterogeneity: Tau² = 15.28; Chi² = 308.35, df = 17 (P < 0.00001); I² = 94%								
Test for overall effect: Z = 11.41 (P < 0.00001)								
**8.3.2 Phacocanaloplasty**								
Arthur 2014	18.2	5.1	32	12.1	3.1	22	3.9%	6.10 [3.91, 8.29]
Lewis 2011	23.5	5.2	30	13.6	4.1	27	3.8%	9.90 [7.48, 12.32]
Lopes-Cardoso 2013	19.8	6.8	24	11	4.2	2	2.4%	8.80 [2.37, 15.23]
Matlach 2013	28.3	4.1	19	12.6	2.1	15	3.9%	15.70 [13.57, 17.83]
Matthaei 2011	17.5	4.2	13	12.8	1.9	5	3.7%	4.70 [1.87, 7.53]
Rekas 2015	19	6.9	29	12.6	2.7	29	3.7%	6.40 [3.70, 9.10]
Schoenberg 2015	19.5	5.7	36	14.1	4.4	36	3.8%	5.40 [3.05, 7.75]
**Subtotal (95% CI)**			**183**			**136**	**25.1%**	**8.14 [4.83, 11.46]**
Heterogeneity: Tau² = 17.49; Chi² = 65.83, df = 6 (P < 0.00001); I² = 91%								
Test for overall effect: Z = 4.82 (P < 0.00001)								
**8.3.3 Canloplasty+Phacocanaloplasty**								
Alobeidan 2015	19.04	6.757	105	15.155714	7.13	105	4.0%	3.88 [2.01, 5.76]
Fujita 2011	23.4	5.5	11	15	4.1	11	3.2%	8.40 [4.35, 12.45]
Rekas 2016	19.1	3.4	10	14.2	3	9	3.7%	4.90 [2.02, 7.78]
**Subtotal (95% CI)**			**126**			**125**	**10.8%**	**5.18 [2.91, 7.46]**
Heterogeneity: Tau² = 2.00; Chi² = 3.94, df = 2 (P = 0.14); I² = 49%								
Test for overall effect: Z = 4.47 (P < 0.00001)								
**Total (95% CI)**			**1498**			**1146**	**100.0%**	**9.94 [8.42, 11.45]**
Heterogeneity: Tau² = 14.11; Chi² = 405.72, df = 27 (P < 0.00001); I² = 93%								
Test for overall effect: Z = 12.89 (P < 0.00001)								
Test for subgroup differences: Chi² = 16.53, df = 2 (P = 0.0003), I² = 87.9%								

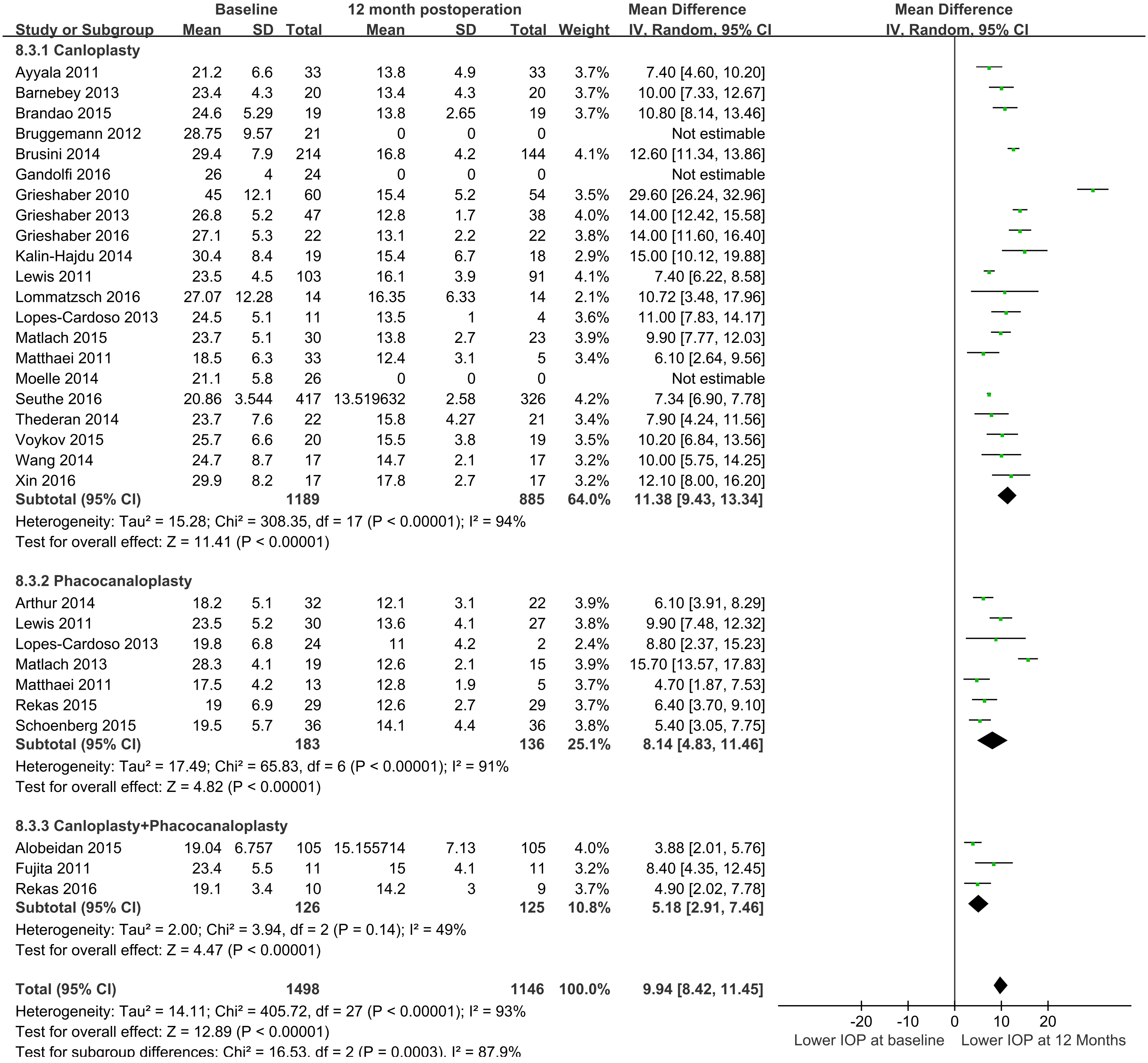

S Fig.2 IOP reduction in canaloplasty at 12 months after surgery

	Baseline			6 month postoperation				Mean Difference
**Study or Subgroup**	**Mean**	**SD**	**Total**	**Mean**	**SD**	**Total**	**Weight**	**IV, Random, 95% CI**
**8.2.1 Canloplasty**								
Ayyala 2011	2.48	0.82	33	2.27	1.83	32	5.2%	0.21 [-0.48, 0.90]
Barnebey 2013	2.2	1.2	20	0	0.001	20	5.7%	2.20 [1.67, 2.73]
Brandao 2015	2.47	0.77	19	0	0	0		Not estimable
Bruggemann 2012	2.9	1	21	0	0.001	21	6.0%	2.90 [2.47, 3.33]
Brusini 2014	3.3	0.9	214	0	0	0		Not estimable
Gandolfi 2016	2.7	3.3	24	0	0	0		Not estimable
Grieshaber 2010	0	0	0	0	0	0		Not estimable
Grieshaber 2013	2.8	0.5	47	0	0	0		Not estimable
Grieshaber 2016	2.9	0.6	22	0	0	0		Not estimable
Kalin-Hajdu 2014	3.7	0.8	19	0.7	1.5	19	5.0%	3.00 [2.24, 3.76]
Lewis 2011	1.9	0.8	103	0.4	0.7	86	6.4%	1.50 [1.29, 1.71]
Lommatzsch 2016	2.7	1.1	14	0	0	0		Not estimable
Lopes-Cardoso 2013	3.4	0.5	11	0.4	0.9	8	5.2%	3.00 [2.31, 3.69]
Matlach 2015	2.6	1.6	30	0.8	1	24	5.2%	1.80 [1.10, 2.50]
Matthaei 2011	2.3	1.2	33	1.5	1.4	23	5.2%	0.80 [0.10, 1.50]
Moelle 2014	2.5	1.8	26	0.2	0.6	25	5.1%	2.30 [1.57, 3.03]
Seuthe 2016	3.456835	0.9	417	0	0	0		Not estimable
Thederan 2014	3.1	1.2	22	0.56	1	22	5.3%	2.54 [1.89, 3.19]
Voykov 2015	3.4	0.5	20	0	0	0		Not estimable
Wang 2014	2.4	1.7	17	0	0	0		Not estimable
Xin 2016	2.9	0.9	17	1.1	1.3	17	5.0%	1.80 [1.05, 2.55]
**Subtotal (95% CI)**			**1129**			**297**	**59.3%**	**2.01 [1.51, 2.50]**
Heterogeneity: Tau² = 0.60; Chi² = 90.34, df = 10 (P < 0.00001); I² = 89%								
Test for overall effect: Z = 7.95 (P < 0.00001)								
**8.2.2 Phacocanaloplasty**								
Arthur 2014	1.3	0.7	32	0.1	0.3	26	6.3%	1.20 [0.93, 1.47]
Lewis 2011	1.5	1	30	0.1	0.3	25	6.1%	1.40 [1.02, 1.78]
Lopes-Cardoso 2013	3.3	0.5	24	0.2	0.6	11	6.0%	3.10 [2.69, 3.51]
Matlach 2013	2.8	1.1	19	0.9	1.3	17	4.9%	1.90 [1.11, 2.69]
Matthaei 2011	2.5	1.3	13	0.3	0.7	9	4.7%	2.20 [1.36, 3.04]
Rekas 2015	2.64	0.68	29	0.22	0.58	29	6.2%	2.42 [2.09, 2.75]
Schoenberg 2015	0	0	0	0	0	0		Not estimable
**Subtotal (95% CI)**			**147**			**117**	**34.4%**	**2.03 [1.36, 2.70]**
Heterogeneity: Tau² = 0.62; Chi² = 76.51, df = 5 (P < 0.00001); I² = 93%								
Test for overall effect: Z = 5.97 (P < 0.00001)								
**8.2.3 Canloplasty+Phacocanaloplasty**								
Alobeidan 2015	2.567143	1.332	105	0.316667	0.78	105	6.3%	2.25 [1.96, 2.55]
Fujita 2011	2.8	0.6	11	0	0	0		Not estimable
Rekas 2016	2.2	1.1	10	0	0	0		Not estimable
**Subtotal (95% CI)**			**126**			**105**	**6.3%**	**2.25 [1.96, 2.55]**
Heterogeneity: Not applicable								
Test for overall effect: Z = 14.94 (P < 0.00001)								
**Total (95% CI)**			**1402**			**519**	**100.0%**	**2.03 [1.69, 2.37]**
Heterogeneity: Tau² = 0.47; Chi² = 172.50, df = 17 (P < 0.00001); I² = 90%								
Test for overall effect: Z = 11.57 (P < 0.00001)								
Test for subgroup differences: Chi² = 0.88, df = 2 (P = 0.64), I² = 0%								

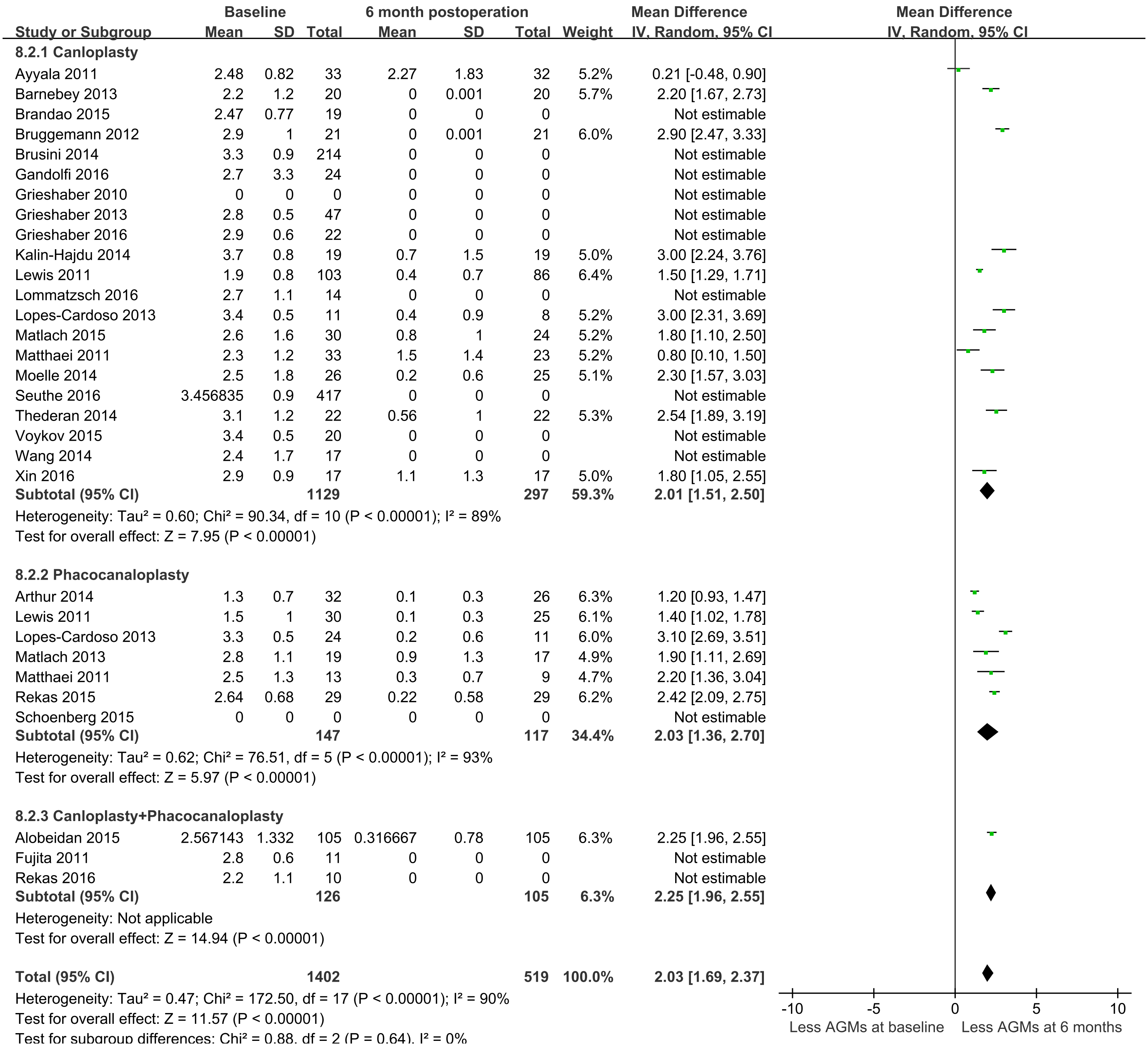

S Fig.3 Anti-glaucoma medications (AGMs) reduction in canaloplasty at 6 months after surgery

Study or Subgroup	Baseline Mean	Baseline SD	Baseline Total	12 month postoperation Mean	12 month postoperation SD	12 month postoperation Total	Weight	Mean Difference IV, Random, 95% CI
**8.4.1 Canloplasty**								
Ayyala 2011	2.48	0.82	33	0.64	0.92	33	4.9%	1.84 [1.42, 2.26]
Barnebey 2013	2.2	1.2	20	0	0.001	20	4.7%	2.20 [1.67, 2.73]
Brandao 2015	2.47	0.77	19	0	0	0		Not estimable
Bruggemann 2012	2.9	1	21	0	0	0		Not estimable
Brusini 2014	3.3	0.9	214	0.7	1.2	144	5.2%	2.60 [2.37, 2.83]
Gandolfi 2016	2.7	3.3	24	0	0	0		Not estimable
Grieshaber 2010	0	0	0	0	0	0		Not estimable
Grieshaber 2013	2.8	0.5	47	0	0	0		Not estimable
Grieshaber 2016	2.9	0.6	22	0.05	0.2	22	5.2%	2.85 [2.59, 3.11]
Kalin-Hajdu 2014	3.7	0.8	19	0.6	1.4	18	4.1%	3.10 [2.36, 3.84]
Lewis 2011	1.9	0.8	103	0.6	0.8	91	5.2%	1.30 [1.07, 1.53]
Lommatzsch 2016	2.7	1.1	14	0	0	0		Not estimable
Lopes-Cardoso 2013	3.4	0.5	11	1	1	4	3.4%	2.40 [1.38, 3.42]
Matlach 2015	2.6	1.6	30	0.8	1.2	23	4.1%	1.80 [1.05, 2.55]
Matthaei 2011	2.3	1.2	33	1.6	1.1	5	3.4%	0.70 [-0.35, 1.75]
Moelle 2014	2.5	1.8	26	0	0	0		Not estimable
Seuthe 2016	3.456835	0.9	417	0.746626	0.955	326	5.3%	2.71 [2.58, 2.85]
Thederan 2014	3.1	1.2	22	0.9	1.3	21	4.1%	2.20 [1.45, 2.95]
Voykov 2015	3.4	0.5	20	1.5	1.6	19	4.1%	1.90 [1.15, 2.65]
Wang 2014	2.4	1.7	17	0	0	0		Not estimable
Xin 2016	2.9	0.9	17	0.9	1.2	17	4.2%	2.00 [1.29, 2.71]
**Subtotal (95% CI)**			**1129**			**743**	**58.1%**	**2.16 [1.79, 2.53]**
Heterogeneity: Tau² = 0.38; Chi² = 152.15, df = 12 (P < 0.00001); I² = 92%								
Test for overall effect: Z = 11.32 (P < 0.00001)								
**8.4.2 Phacocanaloplasty**								
Arthur 2014	1.3	0.7	32	0.1	0.3	22	5.2%	1.20 [0.93, 1.47]
Lewis 2011	1.5	1	30	0.1	0.4	27	5.0%	1.40 [1.01, 1.79]
Lopes-Cardoso 2013	3.3	0.5	24	0	0.001	2	5.3%	3.30 [3.10, 3.50]
Matlach 2013	2.8	1.1	19	1	1.5	15	3.7%	1.80 [0.89, 2.71]
Matthaei 2011	2.5	1.3	13	0.4	0.6	5	3.8%	2.10 [1.22, 2.98]
Rekas 2015	2.64	0.68	29	0.27	0.67	29	5.1%	2.37 [2.02, 2.72]
Schoenberg 2015	0	0	0	0	0	0		Not estimable
**Subtotal (95% CI)**			**147**			**100**	**28.0%**	**2.04 [1.15, 2.92]**
Heterogeneity: Tau² = 1.15; Chi² = 179.42, df = 5 (P < 0.00001); I² = 97%								
Test for overall effect: Z = 4.49 (P < 0.00001)								
**8.4.3 Canloplasty+Phacocanaloplasty**								
Alobeidan 2015	2.567143	1.332	105	0.199048	0.621	105	5.2%	2.37 [2.09, 2.65]
Fujita 2011	2.8	0.6	11	1.2	0.8	11	4.5%	1.60 [1.01, 2.19]
Rekas 2016	2.2	1.1	10	0.1	0.3	9	4.2%	2.10 [1.39, 2.81]
**Subtotal (95% CI)**			**126**			**125**	**13.9%**	**2.07 [1.59, 2.56]**
Heterogeneity: Tau² = 0.12; Chi² = 5.40, df = 2 (P = 0.07); I² = 63%								
Test for overall effect: Z = 8.35 (P < 0.00001)								
**Total (95% CI)**			**1402**			**968**	**100.0%**	**2.11 [1.80, 2.42]**
Heterogeneity: Tau² = 0.47; Chi² = 338.79, df = 21 (P < 0.00001); I² = 94%								
Test for overall effect: Z = 13.23 (P < 0.00001)								
Test for subgroup differences: Chi² = 0.12, df = 2 (P = 0.94), I² = 0%								

Mean Difference
IV, Random, 95% CI

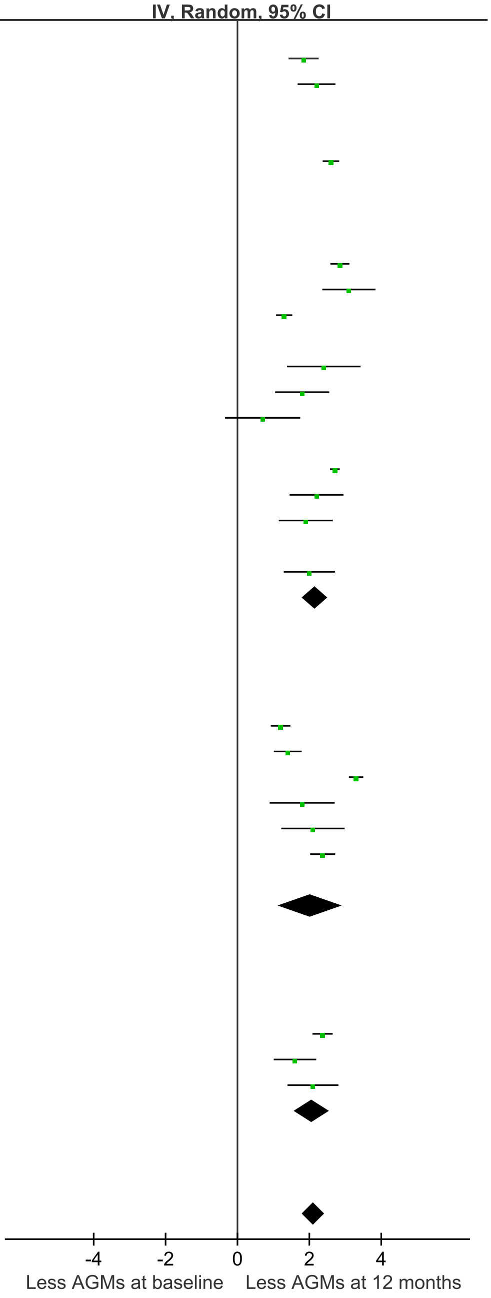

S Fig.4 Anti-glaucoma medications (AGMs) reduction in canaloplasty at 12 months after surgery

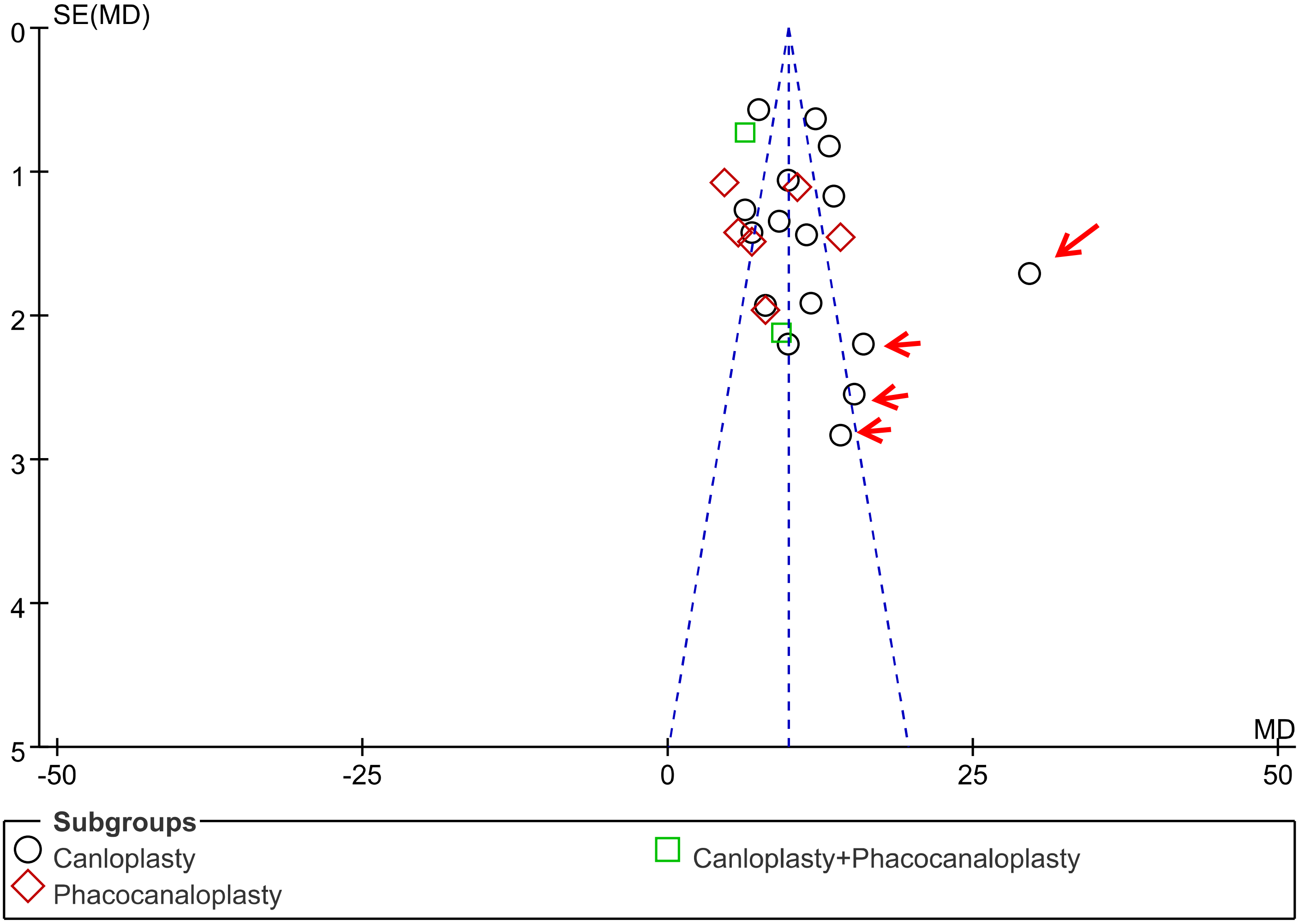

S Fig.5 Funnel plot of IOP reduction in canaloplasty at 6 months after surgery

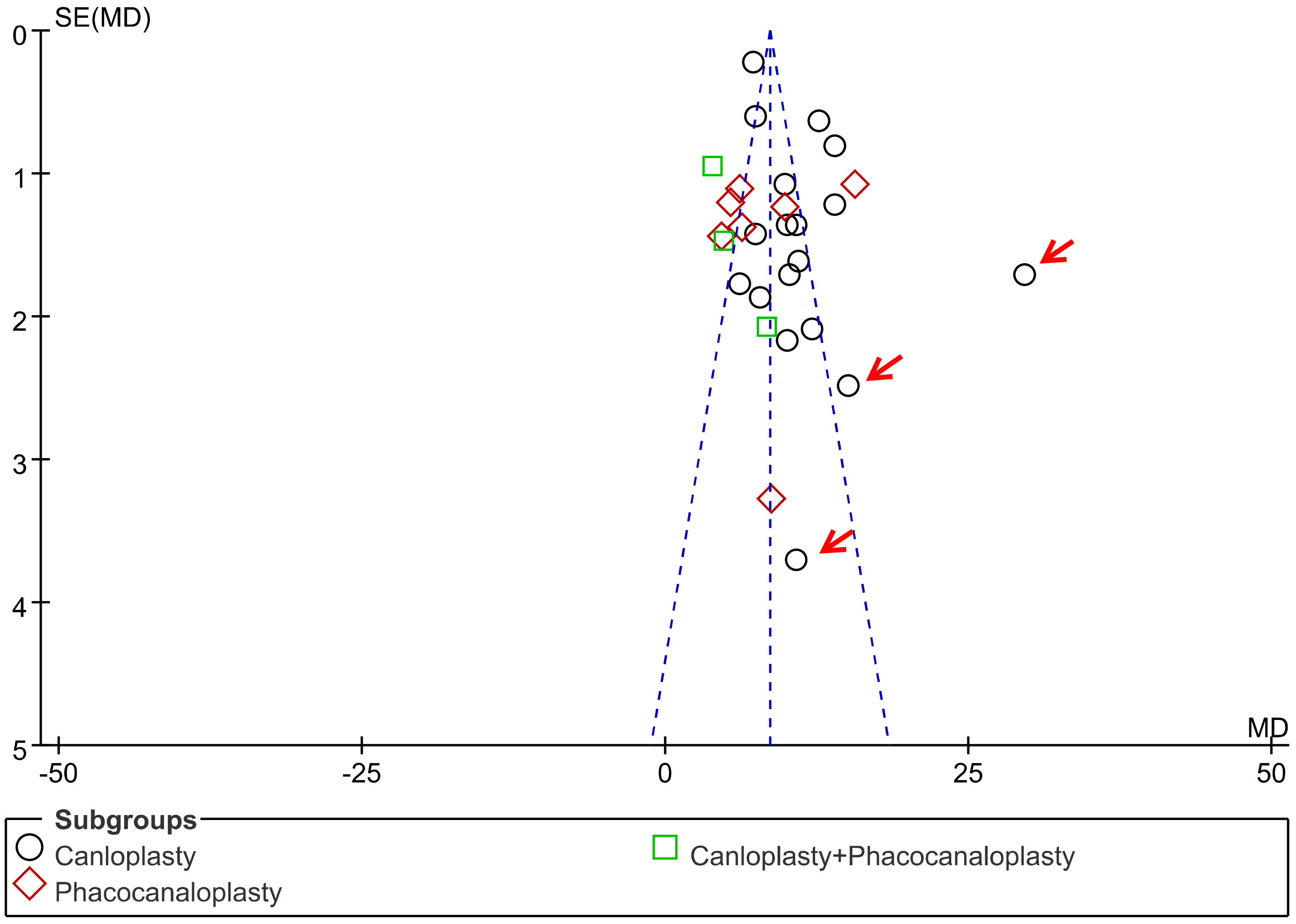

S Fig.6 Funnel plot of IOP reduction in canaloplasty at 12 months after surgery

	IOP reduction mmHg (95% CI), 6 months	AGMs reduction(95% CI),6 months	IOP reduction (95% CI), 12 months	AGMs reduction(95% CI),12 months
Before deleting	10.69(8.96,12.43)	2.03(1.69,2.37)	9.94(8.42,11.45)	2.11(1.80,2.42)
Deleting retrospective studies	12.19(9.60,14.77)	2.14(1.59, 2.68)	11.64(9.07,14.21)	2.22(1.70,2.75)
Deleting small weight studies	10.40(8.07,12.72)	2.11(1.63,2.58)	9.00(7.49,10.51)	2.11(1.69,2.54)

S Table 1 Sensitivity analysis of IOP and anti-glaucoma medications (AGMs) reductions in canaloplasty by removing retrospective studies and small weight studies.

		Ayyala 2011	Bruggemann 2012	Thederan 2014	Matlach 2013	Schoenberg 2015
SELECTION	Case definition adequate	★	★	★	★	★
	Representativeness of cases	★	★		★	★
	Selection of controls					
	Definition of controls	★	★	★	★	★
COMPARABILITY	Comparability (Main outcomes)	★		★	★	★
	Comparability (other(s))	★		★	★	★
EXPOSURE	Cases and controls: same ascertainment method	★	★	★	★	★
	Cases and controls: same nonresponse rate	★	★	★	★	★
	Ascertainment of exposure	★	★	★	★	★

S Table 2 Quality assessment of the retrospective studies in meta-analysis between canaloplasty and trabeculectomy with Newcastle-Ottawa quality assessment scale.

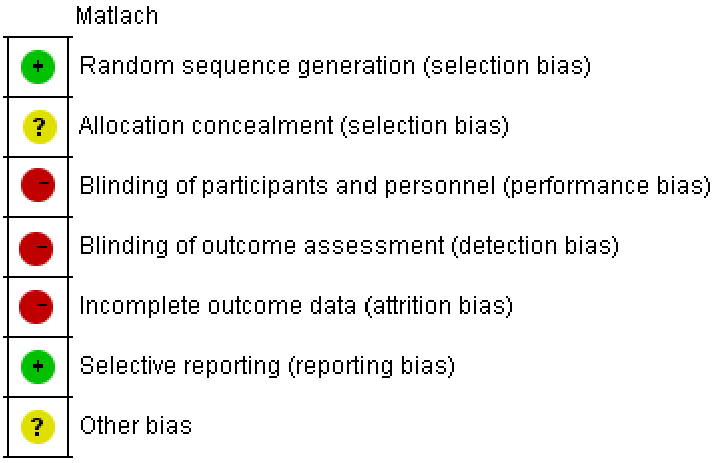

S Fig. 7 Quality assessment of the randomized controlled trial in meta-analysis between canaloplasty and trabeculectomy with risk of bias table.
